# Supplementary material for: A nonlinear decomposition analysis of the rural-urban disparities in tobacco use among women in sub-Saharan Africa
Source: PLoS One. 2025 Sep 9;20(9):e0331738. doi: 10.1371/journal.pone.0331738 (PMC12419646; doi:10.1371/journal.pone.0331738)
Supplement: Attachment — Submitted filename: Review of manuscript_PONE-D-24-57996.pdf [file pone.0331738.s001.pdf]

Review of manuscript: A nonlinear decomposition analysis of the rural-urban disparities in tobacco use among women in sub-Saharan Africa

General Comments

The manuscript titled "A nonlinear decomposition analysis of the rural-urban disparities in tobacco use among women in sub-Saharan Africa" addresses an important public health concern by exploring the rural-urban disparities in tobacco use among women in SSA. The study employs a robust multivariate non-linear decomposition analysis and provides meaningful insights into the factors contributing to tobacco use disparities. The findings have significant implications for policymaking and targeted interventions. However, there are some areas that require improvement/corrections to enhance the clarity and consistency of the study's presentation.

Introduction

The introduction is well-structured and provides adequate background information on the topic.

Methods

Page 9, Line 237: "Hence, even if rural residents have **some** level of"- Is it some or same? The authors should verify and correct if this is an error.

Results

a. Page 9, Line 259 and **Page 11, Line 267**: The authors refer to "Figure 1" in the main text. However, the supporting information labels this figure as "Figure 2". This inconsistency could confuse readers. The authors should carefully review and align the figure numbering in both the main text and supporting materials to ensure consistency and clarity.

b. Page 10, line 274-275: "Similarly, being in a female-headed household and having a higher wealth index was associated with lower odds of tobacco use in both rural and urban areas"- According to Table 2, the aOR for the "sex of household head" in urban areas is **1.10** for female-headed household, which indicates that being in a female-headed household is associated with higher odds of tobacco use in urban areas, not lower odds. The authors should re-evaluate this statement and revise it to reflect the results accurately.

c. Page 10, line 279: "reading newspapers or magazines [aOR = 1.22; 95%CI: 1.11, **1.25**]"- In table 2, it is mentioned as [aOR= 1.22; 95% CI: 1.11, **1.35]**. The authors should check this and revise it to reflect the results accurately.

d. Page 10, line 285-286: "tobacco use with women in Southern Africa were more likely to use tobacco [**aOR = 2.73; 95%CI: 2.30, 3.24**] in urban areas" is actually the pooled value for Southern Africa. In table 2, for urban areas it is "[aOR = 3.30; 95%CI: 2.62, 4.15]". The authors should check this and revise it to reflect the results accurately.

e. Page10, line 286-287: "whereas in rural areas, women in Eastern Africa reported

the highest likelihood of tobacco use [**aOR = 3.30; 95%CI: 2.62, 4.15]**" is actually the urban value for Southern Africa. According to Table 2, for rural areas in Eastern Africa it is "[aOR = 3.06; 95%CI: 2.59, 3.62]". The authors should check this and revise it to reflect the results accurately.

Discussion

Page 16, line 373-378: The authors could provide a reference for this statement to support it with evidence.

Page 17, line 414-421: The authors could provide reference for these statements to support with evidence.
