# Supplementary material for: A nonlinear decomposition analysis of the rural-urban disparities in tobacco use among women in sub-Saharan Africa
Source: PLoS One. 2025 Sep 9;20(9):e0331738. doi: 10.1371/journal.pone.0331738 (PMC12419646; doi:10.1371/journal.pone.0331738)
Supplement: Attachment — Submitted filename: Scientific Review of Manuscript PONE-D-24-57996.docx [file pone.0331738.s002.docx]

**Scientific Review of Manuscript PONE-D-24-57996**

**Title:** A Nonlinear Decomposition Analysis of the Rural-Urban Disparities in Tobacco Use Among Women in Sub-Saharan Africa

**Reviewer’s Comments**

| **Sl#** | **Comment** |
| --- | --- |
| 1 | The title accurately reflects the study’s content, but consider specifying "multivariate nonlinear decomposition" for clarity. |
| 2 | The introduction provides a strong rationale for the study, but it could benefit from a clearer articulation of the research gap. What specific aspect of rural-urban disparities has not been addressed in previous research? |
| 3 | The methodology description is clear, but it would be helpful to justify why a nonlinear decomposition approach was chosen over other decomposition methods. |
| 4 | The results mention that differences in characteristics accounted for 167.48% of the gap in tobacco use. This percentage exceeds 100%, which might need further clarification for readers unfamiliar with decomposition analysis. |
| 5 | The conclusion emphasizes the importance of education and wealth index in reducing tobacco use disparities. However, consider briefly discussing potential policy implications in the conclusion section. |
| 6 | The study design and data source are well described. However, it would be beneficial to discuss any limitations of pooling data from multiple countries in SSA, given potential heterogeneity in tobacco control policies. |
| 7 | Statistical analysis is robust, but the rationale for choosing specific covariates in the logistic regression model should be expanded. Were there any omitted variables that could influence tobacco use? |
| 8 | The use of spatial maps to depict tobacco prevalence is an excellent approach. However, the manuscript does not provide information on how spatial variability was statistically tested. Were any spatial regression models considered? |
| 9 | The discussion section is comprehensive, but it would benefit from additional citations comparing findings with studies outside SSA. Are these trends consistent globally? |
| 10 | The limitations section acknowledges key concerns, such as self-reported data and survey year differences. However, there is no mention of potential recall bias or misclassification of tobacco use, which could affect results. |
| 11 | The authorship contributions are well stated, but the manuscript does not mention whether the authors had any conflicts of interest regarding the topic. |
| 12 | The ethics statement is clear, but it would be helpful to discuss how data protection and participant confidentiality were maintained, especially given the sensitivity of tobacco use data. |

**Overall Evaluation:**

- **Strengths:** The study employs a sophisticated analytical method (nonlinear decomposition) to explore an important public health issue. The use of DHS data strengthens the generalizability of findings.
- **Areas for Improvement:** Some methodological aspects need further clarification, particularly in explaining decomposition results that exceed 100%. Additionally, more discussion on policy implications would enhance the manuscript’s impact.
