# Supplementary material for: A nonlinear decomposition analysis of the rural-urban disparities in tobacco use among women in sub-Saharan Africa
Source: PLoS One. 2025 Sep 9;20(9):e0331738. doi: 10.1371/journal.pone.0331738 (PMC12419646; doi:10.1371/journal.pone.0331738)
Supplement: Attachment — Submitted filename: Response to Reviewers Comments_R2.docx [file pone.0331738.s005.docx]

**RESPONSE TO REVIEWERS’ COMMENTS**

Review Comments to the Author

Please use the space provided to explain your answers to the questions above. You may also include additional comments for the author, including concerns about dual publication, research ethics, or publication ethics. (Please upload your review as an attachment if it exceeds 20,000 characters).

Response: Thank you.

**Reviewer #1**: I recommend acceptance of the manuscript for publication, as the authors have adequately and appropriately addressed all of my previous comments. However, I encourage the authors to carefully review the manuscript for any remaining spelling and grammatical errors prior to final publication. For instance, in the track-changed version, there is still a spelling error on page 15, line 354—“uneployed” should be corrected to “unemployed.”

Response: Thank you. The authors have corrected all spelling and grammatical errors in the manuscript.

**Reviewer #2**: Thank you for your effort and addressing the reviewer comments. Hope this paper will add new knowledge in the respective field.

Response: Thank you.
